# Modulation of Immunity and the Inflammatory Response: A New Target for Treating Drug-resistant Epilepsy

**DOI:** 10.2174/157015913804999540

**Published:** 2013-01

**Authors:** Nian Yu, Hao Liu, Qing Di

**Affiliations:** a Department of Neurology, Nanjing Brain Hospital affiliated to Nanjing Medical University, Nanjing, Jiangsu, China;; b Department of Neurology, University of Pittsburgh School of Medicine, Pittsburgh, PA, USA

**Keywords:** Immunity, Inflammation, Drug-Resistant Epilepsy, Auto-antibodies, Cytokines.

## Abstract

Until recently, epilepsy medical therapy is usually limited to anti-epileptic drugs (AEDs). However, approximately 1/3 of epilepsy patients, described as drug-resistant epilepsy (DRE) patients, still suffer from continuous frequent seizures despite receiving adequate AEDs treatment of sufficient duration. More recently, with the remarkable progress of immunology, immunity and inflammation are considered to be key elements of the pathobiology of epilepsy. Activation of inflammatory processes in brain tissue has been observed in both experimental seizure animal models and epilepsy patients. Anti-inflammatory and immunotherapies also showed significant anticonvulsant properties both in clinical and in experimental settings. The above emerging evidence indicates that modulation of immunity and inflammatory processes could serve as novel specific targets to achieve potential anticonvulsant effects for the patients with epilepsy, especially DRE. Herein we review the recent evidence supporting the role of inflammation in the development and perpetuation of seizures, and also discuss the recent achievements in modulation of inflammation and immunotherapy applied to the treatment of epilepsy. Apart from medical therapy, we also discuss the influences of surgery, ketogenic diet, and electroconvulsive therapy on immunity and inflammation in DRE patients. Taken together, a promising perspective is suggested for future immunomodulatory therapies in the treatment of patients with DRE.

## I INTRODUCTION

Affecting around 50 million people worldwide, epilepsy is a most common disabling neurological disorder, which is characterized by recurring unprovoked seizures [1]. Pharmacotherapy remains to be the major approach of antiepileptic therapy till now [2]. Even the first non-folk medication of epilepsy, potassium bromide, has been introduced for one-and-a-half century [3, 4], and the introduction of over 10 new-generation anti-epileptic drugs (AEDs) in the last decades, the currently available AEDs still only provide a satisfactory level of seizure control in up to 70% of patients with epilepsy [5, 6]. Uncontrolled seizures are occurred in nearly 1/3 of adult epilepsy patients, which are defined as drug-resistant epilepsy (DRE) [7]. Meanwhile, about 10-30% of children with epilepsy are also resistant to currently available AEDs [8]. It is astonishing and disappointing to notice that a rate of seizure control of epilepsy was reached 80 years ago [9]. Therefore, drug resistance in epilepsy is equally obvious and prompts a maintained interest towards both the clinical aspects and the biological mechanisms of drug intractability in epilepsy. Moreover, many of the drugs are associated with severe side effects, which include psychiatric side effects, increased risk of suicide [10], and fatal hepatotoxicity [11]. Persistent seizures have negative psychosocial, behavioral, cognitive, and financial consequences and are associated with an increased mortality rate [9-12]. Therefore, one of the main challenges for the current epilepsy therapy is to develop alternative new anticonvulsant approaches. 

The development of epilepsy usually consists of 3 phases [13]: (i) initial precipitation brain damaging, eg, head trauma, stroke, viral encephalitis, status epilepsy (SE); (ii) epileptogenesis, also as a latent (seizure-free) phase; (iii) spontaneous recurrent seizures, a real epilepsy phase. Recently, growing evidence has demonstrated that immune system dysfunction and inflammatory signaling may play an instrumental role in all the phases of the epilepsy development [14-16]. Modulation of immunity and inflammatory response exerted remarkable protective effects on the epilepsy brain in experimental animal studies [17, 18]. Meanwhile, auto-antibodies were detected in the DRE patients and immunotherapy has been shown to benefit these patients [19]. The evidence above offered the promising prospect of new epilepsy therapies. Gaining a better understanding of the reciprocal interaction between the immune system and the epileptic brain is essential to support the full therapeutic potential of the immunology of epilepsy. According to the epidemiology of DRE, this review refers to acquired epilepsies.

This review aims to: i) discuss the role of immunity and inflammation on the development of DRE; and ii) summarize the current approaches of immunomodulation and anti-inflammatory therapy in the treatment of DRE; and iii) analyze the benefits and side effects of these treatments, and to discuss the future directions.

## II POTENTIAL MECHANISMS OF DRE 

In 2010, the International League Against Epilepsy (ILAE) recommended a definition to DRE, which is "failure of adequate trials of at least 2 tolerated and appropriately chosen and used AEDs to achieve sustained seizure freedom" [20]. However, by this definition, the description in fact designates multi-drug resistant epilepsy (MDR), which commonly occurred in DRE patients [21]. 

**Fig. (1):**
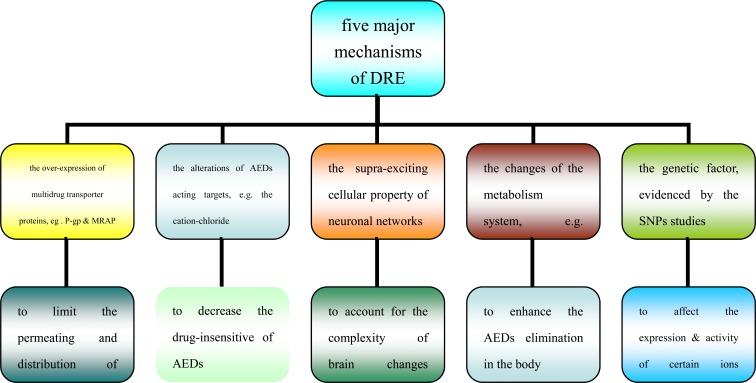
The five major mechanisms of DRE.

At present, there are 5 major hypotheses regarding the mechanisms underlying DRE (see Fig. **1**). The most generally accepted mechanism was the increased activity of blood-brain barrier (BBB) multidrug transporter proteins, such as P-glycoprotein (P-gp) and multi-drug resistance-associated proteins (MRPs) [22], leading to the AED concentration below the effective threshold. However, all the above are relevant but seem insufficient to understand the mechanisms of DRE alone, as neither the transporter, nor the target hypothesis, taken in isolation, can fully explain clinical DRE. Therefore, in some resistant patients, the basic mechanisms above may most likely act in conjunction. 

A successful strategy aimed at overcoming resistance to AEDs necessitates an integrated vision encompassing the basic features of intractable epilepsies. The brain is not immunologically privileged, as it was long considered because of the presence of the BBB and the lack of a conventional lymphatic drainage [23]. Brain is an immunologically specialized site that has its own resident immune system, in which glia cells not only serve supportive and nutritive roles for neurons but also can engage in several inflammatory processes that protect the central nervous system(CNS) from pathogens and enable it to recover from stress and injury [24]. Abnormal glia functions can result in a more severe and chronic neuroinflammatory cycle that promotes or leads neurodegenerative diseases [25]. Recent studies have revealed the involvement of inflammation processes, including over-expression proinflammatoty cytokines, functional glia changes and altered intercellular communication related to gap junctions in the brain of DRE [26, 27]. The evidence for immunological mechanisms in epilepsy can be examined within the following main areas: some childhood epilepsy syndromes, epilepsy associated with other immunologically mediated diseases, and the more common unselected groups of patients with epilepsy [28]. Inflammation is evidenced as an important factor in the pathophysiology of seizure generation and epileptogenesis [29]. Additionally, peripheral inflammation, or systemic inflammation could also induce a mirror inflammatory response in the brain that increased the transient or long-term effects on seizure susceptibility [30]. Furthermore, according to our and others’ recent studies, inflammation and immunity could regulate the expression, activity, and functions of many drug-metabolizing enzymes and drug transporters in the brain of epileptic seizure models [31, 32]. This provides further clues to the future AED discovery targets. 

## III ROLES OF IMMUNE AND INFLAMMATION IN DRE

### A Immunity and Inflammation in Epilepsy: Basic Evidence

Inflammation in the brain is usually caused by an precipitate injurious stimulus of peripheral neurons and results in the release of cytokines and neuropeptides with a variety of cytological and chemical reactions, which further affect the brain vascular permeability and help initiate proinflammatory and immune reactions [33]. The role of the cytokines, inflammatory cells and blood vessels (mainly BBB) will be discussed below.

#### 1 Cytokines and Epilepsy

Pro-inflammatory and anti-inflammatory cytokines, chemokines, and prostaglandins are responsible for the production of an early immune response [34]. Experimental and clinical findings in the past decade have shown that some specific inflammatory mediators and their cognate receptors are up-regulated in epileptic brain tissue [35]. This evidence was corroborated by microarray analysis of various classes of gene transcripts, showing prominently up-regulated pro-inflammatory genes. The most well-known cytokines include interleukin-6 (IL-6), TNF-α and IL-1β [36]. Activation of IL-1 type 1 receptor/Toll-like receptor (IL-1R/TLR) signal pathway has been evidenced as a significant factor contributing to seizure activity in both experimental seizure animal models and in human epileptic tissue from the DRE patients [37]. This signal pathway can be activated by the ligands associated with infections or by endogenous molecules, such as pro-inflammatory cytokines or danger signals [38]. It was reported that IL-1β and high-mobility group box 1 (HMGB1) were simultaneously synthesized and released by astrocytes and microglia in the brain of the mice seizure models. Notably, a rapid release of HMGB1 from neurons appears to be triggered by proconvulsant drugs even before seizure occurrence and is involved in their precipitation of seizures [39]. IL-1 is also involved in the synthesis of IL-6 and TNF-α [40]. The increasing of these cytokines is usually followed by a cascade of inflammatory events that could possibly recruit other cells of the adaptive immune system as a response; the responses are remembered and each time a stronger response is induced [41]. In another study, during amygdala kindling in rat seizure models, systemic administration of TNF-α significantly prolonged the epileptiform discharges and intra-hippocampally injection of anti-inflammatory cytokines IL-10 reduced the duration of primary and secondary after-discharges, without affecting the duration of interictal activity or behavioral seizures [42]. 

Takemiya T. *et al.* [43] found that KA microinjection into brain hippocampus area induced a delayed over-expression of COX-2 in non-neuronal cells, such as endothelial cells and astrocytes. In the injection side, PGE_2_ concentration gradually increases after KA injection, similar to the pattern of non-neuronal COX-2 over-expression. Selective COX-2 inhibitor NS398 treatment abolished this delayed PGE_2_ elevation, as well as blocked hippocampal cell death. Moreover, COX-2 knockout mice are also resistant to neuronal death after KA treatment. Pretreatment with the COX-2 inhibitor restored the anticonvulsant activity of phenobarbital in rats that failed to exhibit a relevant response before celecoxib treatment [44]. However, endogenous IL-1β may also possess anticonvulsive properties, which may be mediated by arachidonic acid metabolites derived from the catalytic action of COX-2 [45]. 

Patients with DRE displayed a pro-inflammatory profile of plasma cytokines without any evidence of increased production from peripheral blood mononuclear cells [46]. These results suggest that the most likely origin for these cytokines is the brain, where cytokines can exert neuromodulatory functions. Our recent meta analysis showed that pro-inflammatory cytokine profile-high IL-6 and low IL-1R antagonist(IL-1Ra) was highly increased in the plasma from patients with epilepsy [47]. Hirvonen J. *et al* found a marker of inflammation-translocator protein, was increased not only *in vitro* in surgical samples from patients with TLE, but also in the seizure focus of living TLE patients [48]. 

Several mechanisms of inflammatory mediators may underlie the recurrence seizure of DRE as follows:

1. Pro-inflammatory cytokines can reduce astrocytic glutamate reuptake by inhibiting astrocytic glutamine synthetase and increase the extracellular glutamate concentration by inducing glutamate release [49]. In particular, the production of PGE_2_ induced in astrocytes by TNF-α upon its release from microglia, mediates astrocytic Ca^2+^-dependent glutamate release [50];
2. The cytokines can rapidly alter the function of classical neurotransmitters by modulating their receptor assembly and phosphorylation at neuronal membranes [51]. The activation of IL-1R/TLR signaling mediates rapid post-translational changes in N-methyl-d-aspartate(NMDA)-gated inward Ca^2+^ channels in pyramidal neurons. IL-1Rs are colocalizes with NMDA receptors on dendrites of neurons [52];
3. Inflammatory mediators can also increase vascular permeability and promote angiogenesis [53]. Thus, their overexpression in perivascular astrocytes and endothelial cells after epileptogenic challenges may affect BBB properties, consequently promoting excitability in surrounding neurons [54];
4. Inflammatory mediators are also critically involved in several different cascades mediating cell death and neurogenesis, as well as synaptic reorganization (i.e. *via *NF-κB activation of chemokines, adhesion molecules and growth factors, free radicals), that are concomitant phenomena of the epileptogenic process in several animal models and human conditions, including post-traumatic epilepsy [55, 56];
5. The cytokines could decrease the seizure threshold in long-term, possibly mediated by transcriptional activation of genes contributing to molecular and cellular plasticity [57];
6. Pharmacological targeting of these pro-inflammatory pathways using selective receptor antagonists, or the use of transgenic mice with perturbed cell signaling, demonstrated that the activation of IL-1R and TLR4 by endogenous IL-1β and HMGB1, is implicated in the precipitation and recurrence of experimentally induced seizures in rodents [38]. This evidence highlights a new target system for pharmacological intervention to inhibit seizures by interfering with mechanisms involved in their genesis and recurrence. It had been observed that IL-1Ra, which was a naturally occurring antagonist to IL-1β, inhibited IL-1β expression in mice astrocytes and decreased seizures in the mice [58]. The anti-inflammatory cytokines were associated with reduction of neuronal cell loss, decreased microglia activation and less BBB leakage [59]. Their proconvulsant activity is hypothesized to be mediated by increased glutamatergic neurotransmission.

#### 2 Auto-antibodies and Epilepsy

In recent years, the detection of auto-antibodies has contributed to the etiologic understanding of a substantial number of unexplained epilepsies, defined as autoimmunity epilepsy [60]. DRE is also associated with the presence of high levels of these antibodies. In the patients with autoimmunity epilepsy, the phenomenon of their resistant to AEDs often showed at the seizure onset [61]. They are usually responded only to anti-inflammation and immunotherapy. Therefore, once the diagnosis has been established the initiation of immunotherapy should be undertaken without delay. However, the full spectrum of autoantibody-associated epilepsies remains to be completely determined. 

At present, the antibodies most relevant to epileptology are those directed to molecules on the surface of neurons, namely to components of the voltage-gated potassium channel (VGKC) complex and to the NMDA-receptor (NMDAR); other antigenic targets located on the neuron surface include the GABA receptor and the α-amino-3-hydroxy-5-methyl-4-isoxazolepropionic acid (AMPA)-receptor [62]. In addition, auto-antibodies can also target some intracellular antigens such as enzyme glutamic acid decarboxylase (GAD) and the onconeural antibodies. Even the precise pathogenic effects of autoantibodies still need to be elucidated, most researchers think that these antibodies are markers of the immunopathological process rather than being pathogenically activated by themselves [63].

The presence of the above antibodies in the CNS was the primary clue to the autoimmune nature of some epilepsies [64]. A promising example for this incipient expansion of the clinical spectrum is the description of a novel seizure type found in patients with antibodies to the VGKC complex [65]. VGKCs are widely expressed throughout the entire CNS. These channels play a vital role in establishing the resting membrane potential and generation of neuronal action potentials, which was importantly related to the seizure onset [66]. 

NMDAR antibody encephalitis is a recently described immunotherapy-responsive panencephalitis with characteristic features that include a psychiatric onset and a later movement disorder [67]. GluR3, one subtype of antibodies to NMDA, was detected in the patients of 3 severe epilepsies: RE, noninflammatory focal epilepsy and "catastrophic" epilepsy [68-70]. With the development of the NMDAR antibody assay, now available worldwide, a few patients with classical limbic encephalitis(LE) and early psychosis and epilepsy have also been found to harbor these antibodies [71]. Antibody levels correlate with the clinical severity of the disease in individual patients, and the antibodies have been shown to substantially reduce NMDAR on hippocampal neurons both *in vitro* and *in vivo*, supporting the likely direct pathogenicity of the NMDAR antibodies [72].

Recognition of autoimmune encephalopathies and epilepsies in children and teenagers with acute or subacute onset of CNS dysfunction, through detection of the pertinent antibody on serum or cerebral spinal fluid, or through a response to immunotherapy may lead to an early diagnosis, and thus expedited implementation of immunotherapy and improved neurological outcome [73]. The epidemiology of pediatric autoimmune encephalopathy and epilepsy is not well established, but advances in disease-specific biomarker discovery have lead to identification of disorders with either a cytotoxic T cell mediated pathogenesis or possible autoantibody mediated disorders [74].

Modoni A. *et al* [75] reported a case of acute nonherpetic LE with negative testing for antibodies directed against onconeuronal and cell membrane antigens, including VGKCs and NMDAR, that showed a dramatic response to treatment with intravenous immunoglobulin (IVIG) followed by a short course of oral prednisone, obtaining a full clinical recovery. This confirms previous observations of "seronegative" autoimmune acute nonherpetic LE, suggesting the presence of other, still unknown central nervous system antigens representing a target of a post-infectious, autoimmune response in these patients. Moreover, it emphasizes the importance of early recognition and treatment of acute autoimmune LE, to reduce the risk of intensive care unit-related complications and the occurrence of permanent cognitive or behavioral defects [75]. 

#### 3 Inflammatory Cells and Gap Junctions

In the immunity and inflammatory response associated with epilepsy, the active cells include the microglia (the resident macrophages of the brain), the astrocytes and the neurons, which are only marginally or not at all involved by endotoxemia [76]. Microglia is part of a major class of glial cells and are a part of the brain’s immune system [77]. Glial cells monitor for signals from brain damage, such as that caused by seizures. Astrocytes are a major player in inflammation of the CNS and are thought to create a balance between endothelial stability and the permeability of the BBB [78]. 

Gliosis(glia extensive proliferation), and astrocytosis (astrocyte proliferationis) are very prominent in the sclerotic hippocampus of the epilepsy patients, particularly in the epileptogenic focus of mesial TLE [79, 80]. The phenomenon above is not only associated with inflammatory processes but also with alterations in astrocytic properties that impact on the DRE state [81]. Quite remarkably, in epileptic patients whose intracranial encephalographies showed several spontaneous seizures originating in the hippocampus, which was subsequently resected, the proportion of seizures was directly correlated with glia density in the hippocampal CA3 area, and electrical seizure duration was directly correlated with the glia density in CA2 and CA3 areas, whereas hippocampal neuronal counts did not correlate with any EEG variables [130]. Glia cells, particularly the astrocytes, may play a key role in the glutamate overflow in mesial TLE [82]. Likewise, functional alterations of specific glia membrane channels, receptors and transporters in sclerotic hippocampi resected from drug-resistant TLE patients have been described [83]. All these data illustrate the current rise of glia, particularly of astrocytes, from the outdated status as a silent majority of brain cells to that of active communication partners with the neurons. 

Gap junctions (GJs) are specific cellular component which contact between eukaryotic cells and provide a direct intercellular communication, as they allow small molecules to be directly exchanged between adjoining cells [84]. GJs are present in nearly all mammalian tissues, the most ubiquitous being Cx43. At least 11 of the 21 human Cx genes are expressed in the nervous system, with different cell specificity GJ intercellular communication is particularly extended among glia, but GJs are also abundantly present in neurons [85]. An elevated level of Cx43 mRNA has been shown in samples of temporal lobe neocortex, surgically resected for DRE, whereas much lower levels of this mRNA were detectable in peri-tumoural temporal lobe tissue samples obtained during removal of cerebral tumours [86]. Similar changes but with less dramatic difference were observed for the mRNA of Cx32, suggesting an increase in the synthesis of GJ protein that may lead to an increase in intercellular coupling, in the temporal cortex of patients exhibiting seizure disorders. Gap-junction blockers applied focally are effective at suppressing seizures and, as such, represent a potential new treatment for epilepsy [87]. Subsequently, it was noticed that the marked astrocytosis in mesial TLE-affected tissues is accompanied by a highly significant increase in astrocytic Cx43 protein levels, suggesting that the large upregulation in Cx43 may exacerbate generalised seizures in the progression of mesial TLE. Various GJ blockers have repeatedly been reported to have anticonvulsant effects in a variety of animal models of epilepsy, including models of DRE, as well as in brain slice models of epilepsy *in vitro* [88]. The involvement of inflammatory processes and alterations in glia functions and in GJ intercellular communication in the DRE are representative, but in no way exhaustive examples, of emergent novel mechanisms for pharmacoresistance in epilepsy [89]. 

#### 4 Brain Blood Barrier

Under normal condition, the BBB serves as a protective structure to the CNS [90]. It prevents the entry of undesirable substances, such as plasma born substances and immune cells [91]. Inflammatory reactions can occur systemically from a damaged BBB or it can be intrinsic. 

In 2008, Fabene *et al.* provided compelling evidence suggesting the key epileptogenic process may actually begin in the BBB [92]. Shortly after SE induced by chemoconvulsant pilocarpine administration, the researchers observed the increases in cell adhesion molecules in brain blood vessels, which are important intermediaries for leukocyte extravasation and adhesion during inflammatory processes. In this study, 4 different leukocyte adhesion molecules—ICAM1, vascular cell adhesion molecule 1 (VCAM1), and selectin E (SELE) and selectin P (SELP) were detected and their levels were up-regulated on the brain endothelium following induction of seizure. The rolling and arresting of the leukocyte were successfully suppressed by antibody blockade of VCAM-1 or SELP and by blockade of their respective leukocyte receptors-integrin α4 subunit of the VLA-4 receptor and SELP ligand, respectively. In addition to decreasing spontaneous seizure frequency and duration, the treatment of α4 integrin–specific antibodies also attenuated cortical and hippocampal cell loss [93, 94]. These findings indicate that seizure activity is associated with leukocytic inflammatory changes in the CNS vasculature. From a clinical standpoint, it is vital to discern whether this pathway holds any therapeutic potential for hyperexcitability. 

The new findings could lead to the repurposing of several anti-inflammation therapeutics on the market or in the clinic, although issues such as safety and applicability to the different types of epilepsy will have to be explored. These findings addressed that blocking the adhesion of leukocytes to endothelium could prevent seizure-induced sequelae and essentially demonstrated that the antibody to integrins had anti-epileptogenic properties. Thus, both anticonvulsant and neuroprotective effects were imparted by these specific antibodies. 

#### 5 Inflammation Induced P-glycoprotein Overexpression

ATP-binding cassette transporters such as P-gp, MRAP, and breast cancer resistance protein are known to transport a wide range of substrates and are highly expressed in the capillary endothelial cells that form part of the BBB [95]. It has been suggested that P-gp overexpression at the BBB lead to therapeutic failure of AEDs by several studies using rodent epilepsy models and human epileptic tissue [96, 97]. Adding a P-gp inhibitor to the anti-epileptic treatment regimens has been shown to reverse the drug-resistant phenotype [98]. The generations of inhibitors of P-gp, such as tariquidar, have been examined in preclinical and clinical studies; however, these trials have largely failed to demonstrate an improvement in therapeutic efficacy [99]. Therefore, interference with the mechanisms that up-regulate P-gp in response to seizure activity might provide a novel alternative approach to control P-gp expression in the epileptic brain to reach better therapeutic effects. 

The other study [100] demonstrated that glutamate could cause localized up-regulation of P-gp *via *COX-2. COX-2 inhibition by SC-58236 and NS-398 may therefore help to increase concentrations of antiepileptic drugs at the target sites in the epileptic brain. However, Holtman L.* et al* [101] reported that the SC-58236 treatment either started before SE or used for 14-day in chronic epileptic rats led to severe adverse effects in the TLE rat model. Therefore, despite a temporal reduction in seizure frequency with SC-58236/PHT treatment, SC-58236 does not seem to be a suitable approach for anti-epileptogenic or anti-epileptic therapy

NF-κB, a major regulator of inflammation, has been linked with epilepsy in many reports [102]. Our recent study [32] found that 24 h after the onset of KA induced seizures, the brain expression of P-gp was significantly increased in the hippocampus CA3 area and amygdala complex, which are important brain regions for epileptogenisis, and this effect was abolished in the rats pretreated with PDTC (pyrrolidine Dithiocarbamic acid ammonium salt), a selective inhibitor of NF-κB by blocking IκB-ubiquitin ligase activity. This data suggested that NF-κB activation played an important role in the up-regulation of P-gp expression in the seizure brain. Our data is consistent with Bauer’s research, which also showed that inhibition of NF-κB by the SN50 peptide could block the inductive effect of the proinflammatory cytokines on P-gp up-regulation in the isolated rat brain capillaries [103]. However, this inhibition did not change the seizure rats’ brain damage and mortality.

P-gp generally drives cellular efflux of a variety of compounds and is thought to be involved in excretion of inflammatory agents from immune cells, like dendritic cells (DCs) [104]. Kooij G. *et al*'s study demonstrated that P-gp could down-modulate DCs function through the regulation of some pro-inflammatory cytokines secretion, resulting in an impaired immune response [105]. Therefore, this work showed a new physiological role for P-gp as an immuno-modulatory molecule and revealed another possible target for immunotherapy.

### B Immunity and Inflammation in Epilepsy: Autoimmune Disorders and Epilepsy

The CNS’s or peripheral system’s autoimmune disorders may involve the possible pathogenesis of DRE [106]. In these conditions, the seizures may be a direct result of the primary disease pathology or could be secondary to the pro-inflammatory processes of these diseases. 

#### 1 Behçet's Disease

Behçet's disease (BD) is a multisystemic, recurrent, inflammatory disorder of unknown etiology [107]. Neurological involvement is characterized either by primary parenchymal lesions or secondary to major vascular involvement. Seizures are not rarely seen in BD and their occurrence can be related to seizure provoking factors or exacerbation of the disease. The seizures were very resistant to AEDs and the epilepsia partialis continua evolving after neurological involvement is also associated with high mortality rate [108]. 

#### 2 Hashimoto Encephalopathy

Hashimoto’s thyroiditis is associated with autoantibodies directed against thyroid peroxidase or thyroglobulin, which leads to the destruction of the thyroid follicular cells and induces hypothyroidism [109]. Although it is usually defined as a corticosteroid-responsive encephalopathy associated with thyroiditis, some patients who suffered from HE, responded only to IVIG therapy. 

#### 3 Neuropsychiatric Systemic Lupus Erythematosus 

Systemic lupus erythematosus (SLE) is an autoimmune disease prevalently affecting any part of the peripheral nervous system or CNS, defined as neuropsychiatric SLE (NPSLE) [110]. The pathogenesis of NPSLE is multifactorial and involves various inflammatory cytokines, autoantibodies, and immune complexes resulting in vasculopathic, cytotoxic and autoantibody-mediated neuronal injury [111]. In one series of 518 consecutive patients with SLE, 88 SLE patients (17%) had epileptic seizures, 60 (11.6%) were considered as primary manifestation of SLE, 23 (4.4%) were secondary acute metabolic causes, and 5 (1%) had epilepsy prior to the diagnosis of SLE [112]. Different treatment regimens include nonsteroidal anti-inflammatory drugs, anticoagulation, and immunosuppressives such as cyclophosphamide, azathioprine, mycophenolate mofetil, and methotrexate. For patients with refractory NPSLE, successful treatments with IVIG, plasmapheresis, and rituximab have been reported [113, 114]. 

#### 4 Multiple Sclerosis

Multiple sclerosis (MS) is a highly prevalent demyelinating disease, often affecting younger female individuals and can result in significant neurologic disability [115]. An autoimmune process is reflected in the development of demyelinating lesions in the white matter. Analysis of the lesions seen on Magnetic resonance imaging that are evident pathologically as MS plaques, reveals inflammatory lymphocytic infiltration consistent with active inflammation, both humoral and cellular [116]. Seizures are a well-described feature and occasionally are the first manifestation of the disease [117]. A recent large meta-analysis of more than19,000 patients with MS revealed that seizures appear in approximately 2-5% of MS patients and often requires medical therapy [118]. Seizures may occur throughout the disease course and may be complicated by either epilepsia partialis continua or generalized tonic-clonic SE. 

## IV IMMUNITY AND ANTI-INFLAMMATORY THERAPIES IN EPILEPSY

The discovery that inflammatory mediators significantly contribute to the onset and recurrence of seizures in experimental seizure models, as well as the presence of inflammatory molecules in human epileptogenic tissue, highlights the possibility of targeting specific inflammation-related pathways to control seizures that are otherwise resistant to the available AEDs [119]. Corticosteroids were first implicated for use in some children epilepsy syndrome. Improvement was also reported in some severe seizures cases secondary to CNS viral infections with the IVIG treatment [120]. Nonsteroidal anti-inflammatory drugs and adrenocorticotropic hormone(ACTH) have also been employed as viable anti-epileptic treatment options. See Fig. (**2**).

### A Glucocorticoids and Associated Drugs

ACTH was firstly used as treatment of DRE in the 1950s, and later, it was found to have astounding results in the treatment of infantile spasms, or West Syndrome(WS). Glucocorticoids (GCs), such as hydrocortisone, are now commonly used in the treatment of some severely drug-resistant childhood epilepsies [121]. The precise mechanism is still unknown, but it is suspected that steroids act on neurotransmitters, such as GABA and glutamate, and thus inhibits seizures [122]. Both GCs and ACTH act as anti-inflammatory mediators and suppresses immune responses [123]. Currently, ACTH and GCs have been successfully used in the treatment of some types of epilepsy, including LGS, myoclonic seizures, Parry Romberg's syndrome, and WS; but their efficacy and safety in treating other types of epilepsy are still need further investigation [124].

Dexamethasone was also of efficacy in controlling pediatric DRE [125]. When dexamethasone treatment preceded exposure to pilocarpine, the number of rats developing SE was reduced [126]. When SE developed, the time-to-onset was significantly delayed compared to pilocarpine alone and mortality associated with pilocarpine-SE was abolished. Dexamethasone also significantly protected the BBB from damage. The effect of 2 additional GCs, methylprednisolone and hydrocortisone, was also reviewed in pediatric drug resistant epilepsy population. Decreased seizure frequency (≥ 50%) or interruption of SE was observed in the majority of the subjects, regardless of the underlying pathology. The mechanism of seizure-reducing effect of dexamethasone may encompass improvement of BBB integrity. 

The conventional steroids using for epilepsy usually have short time effect. Deflazacort, a glucocorticoid used as an anti-inflammatory and immunosuppressant, has shown similar effects to prednisone, but with less adverse effects [127]. Deflazacort should be considered in the therapeutic armamentarium for epileptic encephalopathies. The drug is as effective as hydrocortisone and may be used in therapy for a long period. Use of ACTH or steroids for treatment of seizures in WS and LGS has lead to a decrease in frequency of seizure in some patients, and sometimes up to 6-8 weeks elapsed before seizure freedom was attained [128]. 

Ganaxolone, a synthetic analog of the neurosteroid allopregnanolone that targets the GABA α-receptor [129]. Ganaxolone has completed two Phase II studies for the epilepsy treatment [130]. Progesterone is a neurosteroid that could potentially have immunosuppression effects and contributions to neuronal excitability changes; firing to neurotransmitters are suppressed [131]. Current findings have suggested benefits mostly in females with decreases in spike frequency [132]. 

### B Intravenous Immunoglobulins

Providing a broad immunomodulating effects, IVIG is one of the earliest applications in medicine, starting from the empirical observation of its beneficial effect on seizures and has been implicated for use in epilepsy since 1977 [133]. Another link between epilepsy and immune deficits was noted in the 1980’s treatment with IVIG provided successful results in childhood epilepsies [134]. Improvement was seen in children with severe epilepsy being treated for respiratory infections with IVIG [135]. 

The possible anticonvulsant properties and the ability of IVIG to interfere with the final common pathway of seizures, with a significant increase in seizure threshold, have been demonstrated in different experimental epilepsy model [136]. Although IVIG may represent a valuable resource in some DREs and its effectiveness has important pathogenetic implications, controlled studies with the systematic monitoring of immunological markers are needed to define more precise indications and to optimize the administration protocols [137]. It has been reported that IVIG responds differently in different forms of epilepsies. For example, patients with WS and LGS response very well to the IVIG treatment [138]. In a review article by van Engelen B.G. *et al*, 23% of epilepsy patients achieved complete seizure remission. He reviewed 24 studies with 368 patients; all patients had intractable epilepsy. In addition, children with RE and LKS also gained beneficial results and improvements with IVIG treatment [139]. Billiau A.D. *et al* conducted a study and found that 31% of the studied population demonstrated a beneficial improvement with ≥ 50% reduction of seizure frequency and 23% had a 20-50% seizure reduction [140].

Successful treatment of LKS and the syndrome of continuous spikes and waves during sleep (CSWS syndrome) with IVIG also has been reported. In a prospective study [141], children diagnosed LKS or CSWS syndrome were treated soon after diagnosis with IVIG. No patient had seizures during IVIG treatment and follow-up. Neuropsychological improvement occurred in one child with CSWS syndrome. 

### C Anticonvulsant Activity of Specific Anti-inflammatory Drugs

Growing evidence, obtained in experimental disease models, demonstrated the anticonvulsant activity of specific anti-inflammatory drugs. These studies show that the IL-1β/IL-1Ra system may modulate epileptic activity and contribute to neuronal excitability. Inhibitors of IL-1-converting enzyme/Caspase-1 and antagonists of IL-1β receptors showed strong anticonvulsant activity in seizure models [142], while exogenous IL-1β has pro-convulsive properties. Drugs that block the IL-1β actions had entered clinical trials as potential therapeutics for autoimmune and inflammatory pathologies, and may also have therapeutic potential in epilepsies associated with pro-inflammatory processes in the brain [143, 144]. Resveratrol, a TLR3 antagonist, reduced the frequency of video-monitored spontaneous seizures in rats after daily administration from day1 to day10 after KA-induced SE [145]. This effect was associated with reduction of neuronal cell loss, and inhibition of mossy fibers sprouting. Jiang *et al* [146] also found that pharmacological inhibition of PGE2 receptor subtype EP2 is neuroprotective in pilocarpine model of SE.

### D Antibody Antagonists

There is growing evidence that autoimmune mechanisms play a substantial role in some epilepsy patients. Numerous studies have reported the detection of theoretically relevant serum autoantibodies, and have shown that these antibodies can be epileptogenic [147]. Autoimmune epilepsy offers a mechanism that potentially could be targeted by monoclonal antibodies (mAbs) such as efalizumab or natalizumab, which are already in the market for inflammatory disorders like psoriasis, MS and Crohn’s disease [148]. Raptiva efalizumab, which targets integrin αL, is marketed to treat plaque psoriasis. Tysabri natalizumab, which targets integrin α4, is marketed to treat MS in the EU and MS and Crohn’s disease [149]. Importantly, since the inflammatory mechanism occurs entirely on the blood side of BBB, the mAb would presumably not have to cross the BBB to show efficacy against seizures. At least 2 such antagonists are in the clinic trial. One is bimosiamose (TBC1269), a pan-selectin antagonist and another is YSPSL, a fusion protein of PSGL-1 and human IgG1 acting as a SELP antagonist. Y’s is now considering a collaboration with Constantin and colleagues to evaluate YSPSL in preclinical epilepsy models [150].

### E Immunosuppressant

Three classical immunosuppressant agents have been used, namely cyclosporine A, FK-506 also known as Tacrolimus, and rapamycin [151]. Their mechanisms of action include inhibition of T-ymphocyte activation. Daily systemic injection of cyclosporine A or FK-506 during electrical amygdala kindling prevented the acquisition of stage 5 seizures in rats [152]. However, after drug withdrawal, stimulated animals showed stage 5 seizures, indicating that the treatments failed to inhibit epileptogenesis while providing anticonvulsant effects [153]. Opposite effects were reported by Suzuki *et al* [154], showing that acceleration of PTZ kindling in rats treated with FK-506. Overall, these data indicate that the efficacy of immuno-suppressant in kindling epileptogenesis is still controversial and requires further investigation, possibly using similar treatment protocols in the same kindling models. 

### F Minozac & Miscellaneous: Anti-inflammatory Treatments

Recent experiments established a putative link between injury-induced inflammation, microglia activation, and seizure threshold. Adult mice exposed to traumatic brain injury (TBI), showed a reduced threshold to electroconvulsive shock-induced seizures [155]. This effect was reversed by minozac, an inhibitor of microglia activation and the concomitant production of pro-inflammatory cytokines. This treatment also prevented the ensuing cognitive dysfunctions seen in mice exposed to TBI and experiencing seizures. Treatment with minozac after SE in PN15 rats reversed the enhanced seizure susceptibility to KA; this drug decreased microglia activation and proinflammatory cytokines in hippocampus [156].

### G Plasma Exchange

Plasma exchange is usually used for DRE patients. Immuno-adsorption was reported to be effective in one DRE patient who had detectable antibodies to GluR3; plasma exchange led to interruption of SE on different occasions, reduced seizure frequency and improvement of cognitive functions. Finally, plasma exchange or IVIG evoked good responses of patients with epilepsy-associated Hashimoto's or viral encephalitis, in which antibodies to VGKCs were found [157].

## V PERSPECTIVES

**Fig. (2):**
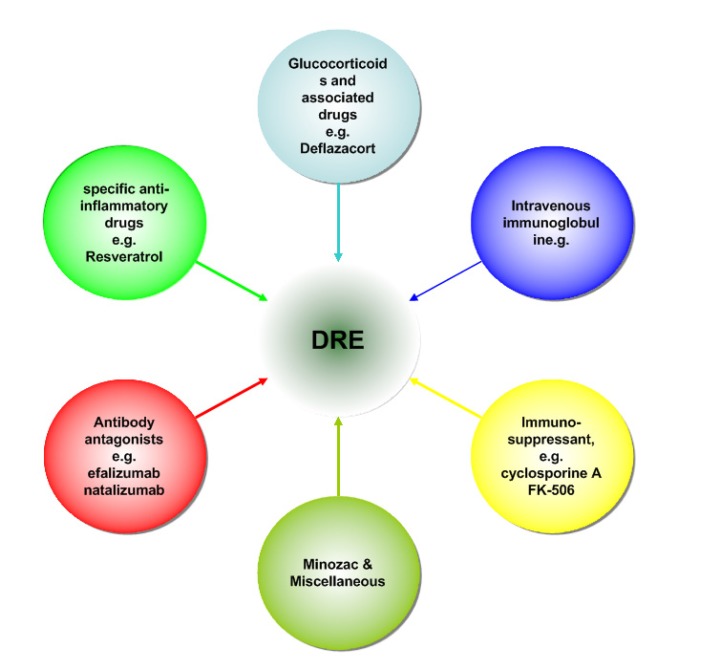
The potential drug targets for anti-inflammatory therapies to treat DRE.

There are several potential links between the immuno-inflammation and the current therapies for DRE (see Fig. **2**). The AEDs have been most intensively investigated on the immune system, as reviewed by Beghi in detail [158]. Much direct evidence related immunity with epilepsy came from the surgical specimens of DRE patients. Surgery has been recommended as the treatment of choice for the DRE patients popularly, but surgery is often delayed for years after people diagnosed of epilepsy, those are very unlikely to improve with further pharmacotherapy [159]. The mechanisms of vagus nerve stimulation(VNS) for epilepsy have not been fully understood. However, anti-inflammatory properties of VNS have been reported in various experimental models of inflammation [160], even it is still unknown whether the anti-convulsive effect of VNS was mediated through this anti-inflammatory mechanism. Meanwhile, many recent studies have shown that ketogenic diet has anti-inflammatory effects and protects against excitotoxicity-mediated neuronal cell death [161, 162]. 

Nowadays deep brain stimulation (DBS) has also be considered as an "alternative choice" in those adult DRE patients with normal intelligence in which neither new AEDs nor resective epilepsy surgery are a reasonable therapeutical option. Curative electroconvulsive therapy (ECT) remains a very useful treatment, still irreplaceable for some specific rare cases especially with a psychiatric history, as long as other brain stimulation methods, such as transcranial magnetic stimulation, remain experimental [163]. DBS could successfully decrease inflammation, which is believed to be the direct source of the pain associated with Parkinson's [164]. The cell transplantation approach is promising in serving as an adept alternative therapy for DRE, because this strategy has shown the capability to curtail epileptogenesis when used soon after an initial precipitating brain injury, and to restrain spontaneous recurrent seizures and improve cognitive function when utilized after the occurrence of DRE [165]. 

Till now, the marketed anticonvulsants fail to inhibit excessive excitatory activity and seizures of all the patients with epilepsy. Immunity and inflammation are an integral part of the pathogenic processes triggered by seizures. The fact that the immune system and inflammation are central to the pathophysiology of epilepsy has raised the prospect of new therapeutic approaches to counteract epilepsy. However, understanding of the crosstalk between the immune system and the DRE brain is still rudimentary and, as suggested by failed clinical trials, not adequate to guide therapeutic interventions. Modulation of adaptive immunity may afford the opportunity to treat DRE. However, immunomodulation can also have deleterious effects that need to be considered. Aspects awaiting further investigations include: (1) the discovery and characterization of novel inflammatory molecules and pathways in multiple DRE models; (2) modifying inflammatory cell and gene therapies represent remote alternatives for which crucial issues still have to be resolved; (3) the validation of inflammatory processes described in the experimental setting in surgically resected epileptogenic tissue from epilepsies of different etiology; (4) development of strategies to target these inflammatory factors in order to inhibit seizures or prevent epileptogenesis, particularly in clinic; (5)what patients are really need non-pharmacological therapeutic strategies, such as surgery, KD, VNS and/or others; and (6) how to bind the non-pharmacological therapeutic strategies above and pharmacological therapies including modulating immunity and inflammation; and (7) much further study will be needed, as there is still some debate whether inflammation directly contributes to the neuronal injury or just simply is a consequence of neuropathogenesis.

Targeting inflammation to treat or even prevent epileptic seizures has multiple advantages compared with targeting neuronal function. First, it avoids side effects of AEDs and it is possible of targeting a cause of the seizures and thus modifying disease. Another advantage would be the prophylactic aspect of the strategy, which could potentially help prevent the development of seizures in patients at risk of epilepsy, including victims of traumatic brain injury, stroke and cerebral infection. The marketed anticonvulsant epilepsy drugs have little value in such a preventative context. Taken together, we believe that further research on anti-inflammatory agents used for the treatment of epilepsy is well warranted and will likely provide exciting possibility for the future of epilepsy treatment.

## Figures and Tables

**Fig. (3):**
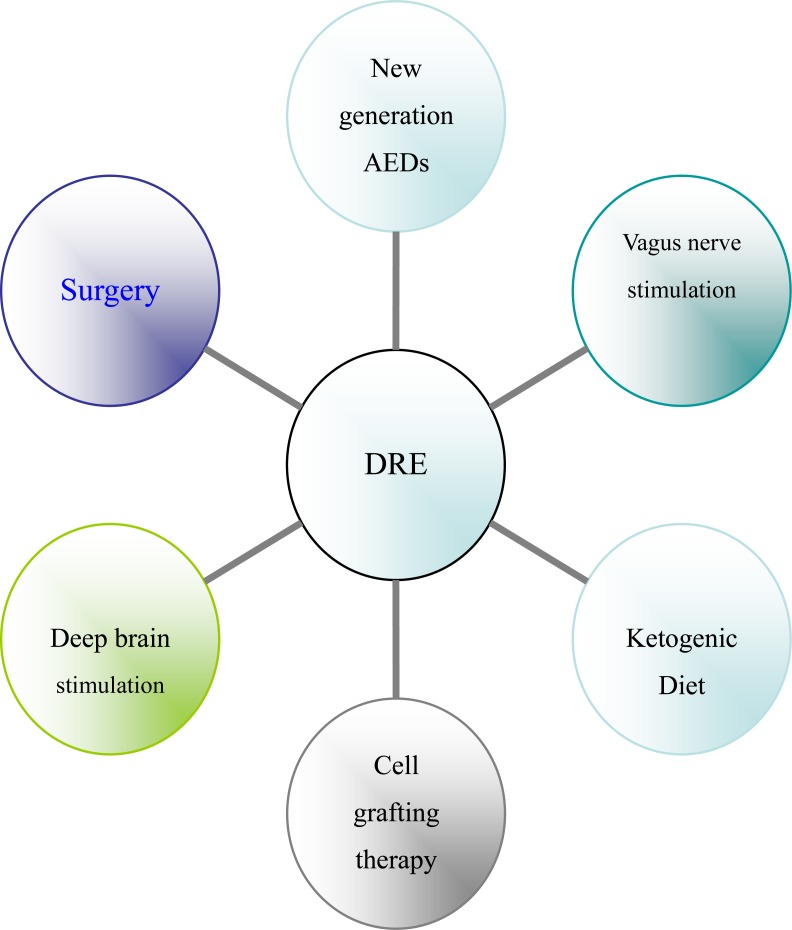
The current therapies for DRE.
